# Bibliography

**Published:** 1894-09

**Authors:** 


### Bibliography.

An Illustrated Dictionary of Medicine, Biology, and Allied Sciences. By George M. Gould, A.M., M.D., author of "The Student's Medical Dictionary;" editor of the *Medical News*, etc. P. Blackiston, Son & Co., Philadelphia. $10; with index, $12.

Dr. Gould has already become well known as the author of dictionaries, but in this great work he has excelled all previous efforts.

Many hundreds of new words have been introduced into medical and dental literature during the past few years, marked as they have been by great scientific activity and progress; and this is largely a fresh gathering from the living literature of the day, and not a mere compilation and copying from the many books already published. It is a stupendous work, in which the author has received valuable assistance from such scholars as Drs. C. W. Green, W. A. N. Dorland, A. A. Eshner, A. C. Wood, S. McClintock Hamill, Emma Billstein, J. C. Da Costa, Professors B. G. Wilder, Charles S. Dolley, S. H. Gage, Henry Leffmann, Albert P. Brubaker, and others, and they have all brought to it the ripest fruits of their knowledge.

This work includes all the more commonly used terms of biology,—a thing highly desirable (1)—as the author states in his preface—because of the modern recognition of the great truth that general biologic science is the foundation of genuine and progressive medical science; (2) because the best schools of medicine are more and more urging or making obligatory the preliminary biologic course of study, and (3) because no satisfactory lexicon of biology exists in English.

In the spelling of certain chemic words the author has followed the advice of the American Association for the Advancement of Science, and has indicated the best pronunciation of words by the simplest and most easily understood phonetic method.

A unique and important feature is the incorporation of a large number of tables, wherein numerous facts are brought together and classified. Among these we may point out tables of acids, arteries, bacteria, electric batteries, foods, hydrocarbons, nerves, operations, paralysis, parasites, points, postures, and tumors. This seems deserving of mention, as there is not to be found in medical literature so complete and digested a *résumé* of surgical operations, of bacteriology, parasitology, tests, tumors, etc., as is here furnished.

A somewhat careful examination of the work has failed to show but a few words upon dental subjects that have been omitted or but what have received just treatment. The labor, patience, and fidelity shown, not only in the author's work but by the publishers and printers, must excite the admiration of every one competent to form, and willing to give, an intelligent opinion. Bearing as it does the stamp of extreme carefulness and accuracy, it will no doubt early take the place of leader among medical dictionaries.

